# Photosynthetic Physiological Characteristics of Water and Nitrogen Coupling for Enhanced High-Density Tolerance and Increased Yield of Maize in Arid Irrigation Regions

**DOI:** 10.3389/fpls.2021.726568

**Published:** 2021-09-23

**Authors:** Yao Guo, Wen Yin, Hong Fan, Zhilong Fan, Falong Hu, Aizhong Yu, Cai Zhao, Qiang Chai, Emmanuel Asibi Aziiba, Xijun Zhang

**Affiliations:** ^1^State Key Laboratory of Aridland Crop Science, Lanzhou, China; ^2^College of Agronomy, Gansu Agricultural University, Lanzhou, China

**Keywords:** maize, irrigation and nitrogen coupling, close planting, photosynthesis, chlorophyll a fluorescence, grain yield

## Abstract

To some extent, the photosynthetic traits of developing leaves of maize are regulated systemically by water and nitrogen. However, it remains unclear whether photosynthesis is systematically regulated *via* water and nitrogen when maize crops are grown under close (high density) planting conditions. To address this, a field experiment that had a split-split plot arrangement of treatments was designed. Two irrigation levels on local traditional irrigation level (high, I2, 4,050 m^3^ ha^−1^) and reduced by 20% (low, I1, 3,240 m^3^ ha^−1^) formed the main plots; two levels of nitrogen fertilizer at a local traditional nitrogen level (high, N2, 360 kg ha^−1^) and reduced by 25% (low, N1, 270 kg ha^−1^) formed the split plots; three planting densities of low (D1, 7.5 plants m^−2^), medium (D2, 9.75 plants m^−2^), and high (D3, 12 plants m^−2^) formed the split-split plots. The grain yield, gas exchange, and chlorophyll *a* fluorescence of the closely planted maize crops were assessed. The results showed that water–nitrogen coupling regulated their net photosynthetic rate (Pn), stomatal conductance (Gs), transpiration rate (Tr), quantum yield of non-regulated non-photochemical energy loss [Y(NO)], actual photochemical efficiency of PSII [Y(II)], and quantum yield of regulated non-photochemical energy loss [Y(NPQ)]. When maize plants were grown at low irrigation with traditional nitrogen and at a medium density (i.e., I1N2D2), they had Pn, Gs, and Tr higher than those of grown under traditional treatment conditions (i.e., I2N2D1). Moreover, the increased photosynthesis in the leaves of maize in the I1N2D2 treatment was mainly caused by decreased Y(NO), and increased Y(II) and Y(NPQ). The coupling of 20%-reduced irrigation with the traditional nitrogen application boosted the grain yield of medium density-planted maize, whose Pn, Gs, Tr, Y(II), and Y(NPQ) were enhanced, and its Y(NO) was reduced. Redundancy analysis revealed that both Y(II) and SPAD were the most important physiological factors affecting maize yield performance, followed by Y(NPQ) and NPQ. Using the 20% reduction in irrigation and traditional nitrogen application at a medium density of planting (I1N2D2) could thus be considered as feasible management practices, which could provide technical guidance for further exploring high yields of closely planted maize plants in arid irrigation regions.

## Introduction

It is estimated that the world population will increase from the current 7.6 to 9.7 billion by 2050, and that higher living standards will require a 70 to 100% increase in the production of major food crops (Godfray et al., 2010; Long et al., 2015). Cultivating new land for the needed crop production is not a viable option, because there is little good quality land available. Hence, securing these types of food will require increased inputs of water and nutrients to fill the gap in yield. Heavy inputs of water and fertilizer, however, can negatively affect marine, freshwater, and terrestrial ecosystems, causing diminished biodiversity and damage to unique habitats (Godfray, 2014). Meanwhile, irrational land reclamation and the extensive management of agricultural production is generating large emissions of greenhouse gases (Burney et al., 2010). To alleviate these pressing problems, global food demands must somehow be met without increasing the amount of arable land, with a focus on increasing grain yield per unit area.

Moderately increasing planting density is the most effective and simple way to achieve high grain yields (Hashemi et al., 2005; Bastos et al., 2020). Generally, a higher planting density will promote mutual shading among neighboring plants, limit the efficiency of interception and utilization of light energy of individual plants (Marchiori et al., 2014), and reduce the photosynthetic rate of leaves, therefore, also affecting crop production (Li et al., 2012, 2015). Research has shown that changes in the light interception environment of mature leaves in the lower canopy layer of crops will affect the structure and photosynthetic characteristics of new leaves in the upper canopy (Jiang et al., 2011). When crops are planted at much higher densities, their photosynthesis is reduced, and yield formation is adversely affected (Wang et al., 2005).

Photosynthesis is not only influenced by planting density but is also regulated by water and nitrogen factors (Yang et al., 2010b; Ghotbi-Ravandi et al., 2015). One of the most important ways to achieve high crop yields is to regulate crop photosynthetic characteristics as well as the accumulation and distribution of photosynthetic products *via* reasonable water and nitrogen management practices (Simkin et al., 2019). Imposing a moderate reduction at irrigation levels can induce associated acclimation processes in crops, such as stimulated root growth, slowed leaf growth, leaf rolling, osmotic activity and the synthesis of protective compounds, and stomatal closure to preserve the normal water content of green leaves (Lopes et al., 2011; Tardieu, 2012). It can also promote the transfer of photosynthetic products into the grain, and enhance the efficiency of crops in using water (Guo et al., 2020). In contrast, with an excess reduction in irrigation, the balance between light interception and energy utilization is disturbed in plants, in which the excessive excitation energy of photosystem II (PSII) can lead to impaired photosynthetic traits and accumulated reactive oxygen species (ROS) (Chaves et al., 2009). Furthermore, minor drought conditions can drive a decline in photosynthesis at different growth stages of crops, but increasing the application of N fertilizer could modulate the effects of limited water on photosynthetic rate and increase the stomatal conductance and transpiration rate of leaves, thereby strengthening the adaptability of plants to adversity under stress conditions (Han et al., 2006). Therefore, reasonable water and nitrogen management practices could be used to regulate the photosynthetic characteristics of crops.

Generally, increasing seeding density alters the light interception intensity and utilization efficiency of crops, and amplifies the competition of their roots for soil water and nutrients (Jensen et al., 2011; Li et al., 2014). Therefore, changes in photosynthetic characteristics, such as pigment content and photosynthetic rate, are maybe due to the combined effects of light interception intensity and competition for soil water and nitrogen when individual crops are planted close to each other. So far, most studies on the systemic regulation of photosynthesis have been conducted under artificially controlled light conditions (Taiz and Zeiger, 2006; Klughammer and Schreiber, 2008; Lambrev et al., 2012). Also, such research often focuses on photosynthetic traits of entire plants under low- or high-light intensity conditions (Li et al., 2010; Deng et al., 2012). Moreover, except for newly developed leaves, the rest of the leaves generally occur in a uniform low light environment (Li et al., 2015). However, in actual field outdoor crop production settings, the light conditions experienced by a densely planted population in the field are more complicated in that the ambient light interception intensity of each leaf gradually improves going from the bottom to the top of the plant (Hu et al., 2016; Wu et al., 2019). There are few studies on whether the photosynthetic systems of densely planted crops is regulated by the interaction of water and nitrogen, and the related regulation mechanism is still unclear. It is conceivable that an innovative system of water and nitrogen management could act to weaken the competition below the ground to promote the photosynthetic performance of a crop above the ground by optimizing the status of soil water and nitrogen when growing at a high density. Further inquiry into this putative mechanism should provide a practical and theoretical basis for improving water and nitrogen management to increase planting density, so as to boost grain yield per unit area.

Maize is a typical C_4_ plant capable of high photosynthetic intensity. Maize plants are widely cultivated around the world, because it is a major grain and fodder crop (Yin et al., 2021). The objectives of this study were to investigate the effects of different water and nitrogen levels on the grain yield of field-grown maize planted at various densities, and to systematically study the effects of different water and nitrogen levels on the photosynthetic performance of field-grown maize planted at various densities. The results of this study will help explain further the photosynthetic physiological basis of water–nitrogen coupling to enhance the high-density tolerance of maize, and lay a foundation for enhancing the potential of water-saving and nitrogen fertilizer reduction in the dense planting of maize in arid irrigated areas of northwest China.

## Materials and Methods

### Study Area

**Figure 1:**
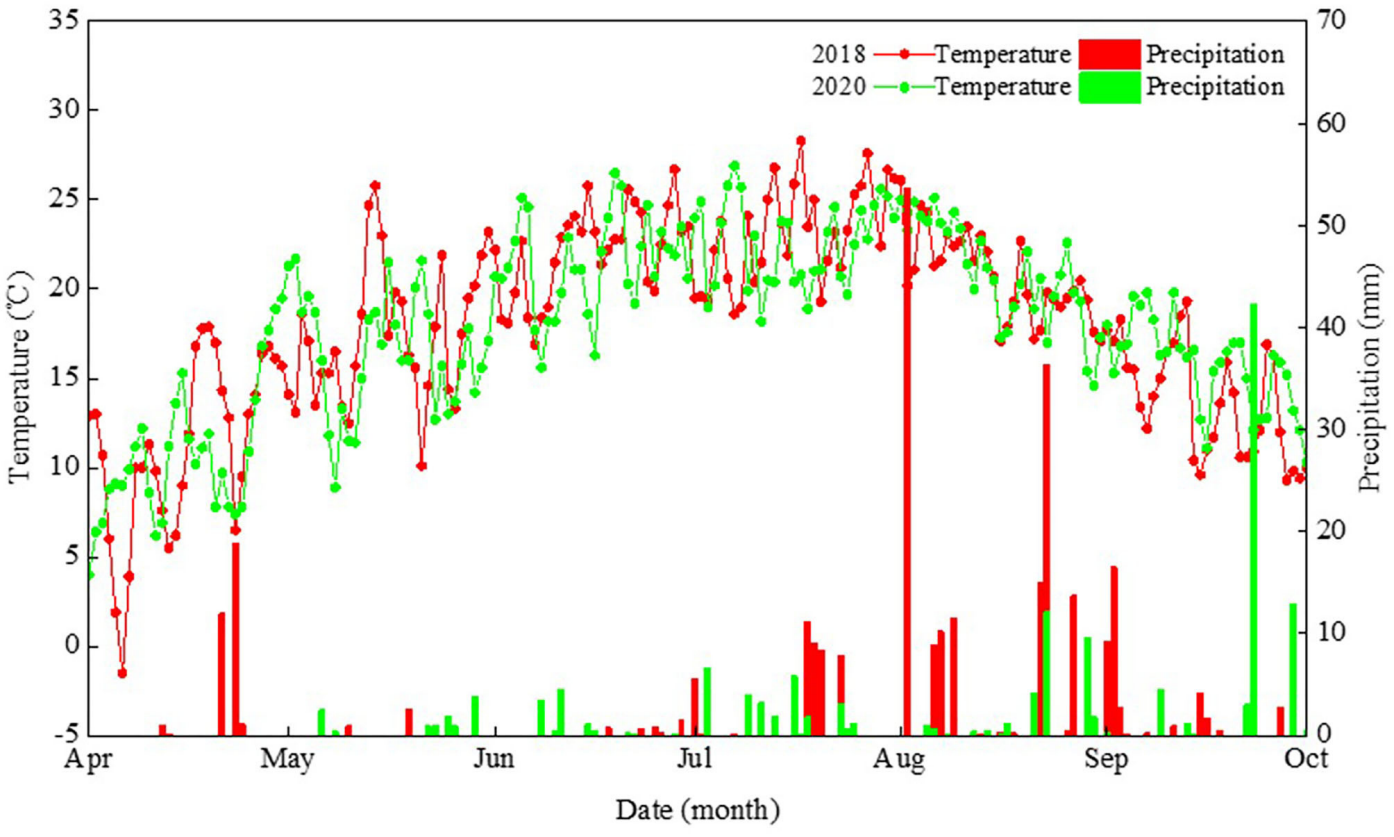
Daily precipitation and mean air temperature during the maize growth period in 2018 and 2020 at the Wuwei Experiment Station in northwestern China.

A field experiment was conducted at the Oasis Agricultural Experimental Station of Gansu Agricultural University, in northwestern China, in 2018 and 2020. The soil of this study region is classified as an Aridisol (FAO-UNESCO, 1988), where the climate type is a cold and temperate arid climate zone. Over the last 50 years, on average, this region has been having annual total solar radiation >5,500 MJ m^−2^, annual sunshine duration >2,800 h, annual accumulated temperature (>10°C) >2,800°C, and an annual frost-free period >155 days. Therefore, the heat and sunshine conditions in the region of study can meet the requirements for field maize cultivation. Here, the planting density for maize is typically 7.5 plants m^−2^ using traditional farming techniques, which is significantly lower than in the maize high-yield field. It is imperative that a cropping system enabling the high yield of maize based on dense planting be developed. The air temperature and precipitation distributions across the growing period of maize in 2018 and 2020 are shown in Figure 1.

### Experimental Design and Plot Management

The field experiment utilized a split-split plot arrangement of treatments in a randomized complete block design. Two irrigation levels, local traditional irrigation level (high irrigation: I2, 4,050 m^3^ ha^−1^) and reduced by 20% (low irrigation: I1, 3,240 m^3^ ha^−1^), formed the main plots. Two pure nitrogen application levels, local traditional nitrogen level (high nitrogen: N2, 360 kg ha^−1^) and reduced by 25% (medium nitrogen: N1, 270 kg ha^−1^) formed the split plot. Three planting densities (low, D1, 7.5 plants m^−2^; medium, D2, 9.75 plants m^−2^; and high, D3, 12 plants m^−2^) formed the split-split plots. Overall, this experiment comprised 12 treatments, each with three replicates (Table 1).

**Table 1:** Description of experimental treatments.

**Irrigation level(m^3^ ha^−1^)**	**Nitrogen (N) fertilizer application (kg N ha^−1^)**	**Planting density (kg N ha^−1^)**		
		**High planting density, 12 plant m^−2^ (D3)**	**Medium planting density, 9.75 plant m^−2^ (D2)**	**Low planting density, 7.5 plant m^−2^ (D1)**
Local conventional irrigation water amount, 4,050 m^3^ ha^−1^(High irrigation, I2)	Local conventional nitrogen amount, 360 kg N ha^−1^ (High nitrogen, N2)	I2N2D3	I2N2D2	I2N2D1
	Local conventional nitrogen amount reduced by 25%, 270 kg N ha^−1^ (low nitrogen, N1)	I2N1D3	I2N1D2	I2N1D1
Local conventional irrigation water amount reduced by 20%, 3240 m^3^ ha^−1^(Low irrigation, I1)	Local conventional nitrogen amount, 360 kg N ha^−1^ (High nitrogen, N2)	I1N2D3	I1N2D2	I1N2D1
	Local conventional nitrogen amount reduced by 25%, 270 kg N ha^−1^ (low nitrogen, N1)	I1N1D3	I1N1D2	I1N1D1

Only nitrogen (urea) and phosphorus (diammonium phosphate) were applied in this experiment as fertilizer, because the potassium content of the soil in this region is sufficiently high to sustain crop growth. In each year, all the experimental plots received the phosphate fertilizer application to maize at a rate of 180 kg P_2_O_5_ ha^−1^, before the maize crops were planted, while maize received 30% of N as base fertilizer at sowing, 50% as topdressing at the big flare opening stage, and the remaining 20% as topdressing at the grain filling stage. In the two studied years, maize cv. *Xian-yu 335*, commonly cultivated in this region, was sown on April 18 and April 20, and harvested on September 28 and September 26, in 2018 and 2020, respectively. Each treatment had a plot dimension of 40 m^2^ (8 × 5 m) and was separated by a 50-cm-wide buffer, with a 50-cm high ridge between the plots to reduce the potential of water spillage. In the experiment, irrigation was carried out using a drip irrigation system in which a flow meter was installed at the discharge end of the pipe to record the amount of irrigation received in each plot. The local traditional irrigation level (I2) was administered at a rate of 900, 750, 900, 750, and 750 m^3^ ha^−1^ at the jointing, big flare opening, silking, flowering, and filling stages, respectively. The local traditional irrigation level reduced by 20% (I1) was likewise administered at similar growing stages of maize.

### Data Collection

#### Determination of Grain Yield

Grain yield was measured in the physiological maturity period of maize. Four rows and non-sampled maize plants with a 3-m length were harvested to determine the grain yield of maize in each treatment. These grains were sun-dried, cleaned, and weighed after threshing with a small grain sheller.

#### Chlorophyll Relative Content

For maize leaves, their chlorophyll relative content (SPAD) was measured using a handy plant chlorophyll meter (SPAD-502; Minolta Camera Co., Ltd., Osaka, Japan). The value of SPAD was determined on three ear leaves between 9:00 and 11:30 a.m. on a sunny day, at the maize silking stage (Li et al., 2015).

#### Gas Exchange and Light Intensity

The net photosynthetic rate (Pn), stomatal conductance (Gs), and transpiration rate (Tr) were measured using a portable photosynthesis system (LI- 6800XT; LI-COR, Lincoln, NE, United States). Measurements were taken on a sunny day in the morning (9:00–11:00 a.m.) to avoid potential stomatal closure around noontime. Measuring time, date, and the position of the leaves were consistent with the values of SPAD obtained for maize (Li et al., 2015).

#### Chlorophyll a Fluorescence

The parameters for chlorophyll *a* fluorescence in the middle part of ear leaves were determined with a portable fluorescence measuring system (PAM-2500; Heinz Walz, Effeltrich, Germany), using the saturation pulse method after 11:00 p.m. to the leaves that were well-adapted to the dark. All the measurements were conducted under darkness. At least three maize leaves from each treatment were measured at the silking stage. Measuring light was set at 1 μmol (photon) m^−2^s^−1^ (to determine the minimum fluorescence yield, F_0_), and light intensity was set at 600 μmolm^−2^s^−1^ to determine maximum fluorescence (Fm'), initial fluorescence (F0'), and steady-state fluorescence (F). Using these data, Fv/Fm, Y (II), NPQ, Y(NPQ), and Y(NO) were then calculated as follows (Klughammer and Schreiber, 2008):

(1) maximal photochemical efficiency of PSII: Fv/Fm = (Fm–F_0_)/Fm,
(2) actual photochemical efficiency of PSII: Y(II) = (Fm'–F)/Fm',
(3) dissipation of excess energy: NPQ = (Fm–Fm')/Fm',
(4) quantum yield of regulated non-photochemical energy loss: Y(NPQ) = F/Fm'–F/Fm, and
(5) quantum yield of non-regulated non-photochemical energy loss: Y(NO) = F/Fm.

### Data Analysis and Statistical Methods

All the experimental data were analyzed with the SPSS 22.0 software (IBM, Armonk, NY, United States) by three-way ANOVAs. Irrigation, nitrogen, density, and their interactions were treated as fixed factors, and replication was considered as a random factor. Differences in means among the treatments were considered significant at *P* < 0.05 according to Duncan’s multiple-range test. Redundancy analysis (RDA) was performed to identify correlations between grain yield and various photosynthetic physiological parameters of maize.

## Results

### Yield Response

**Figure 2:**
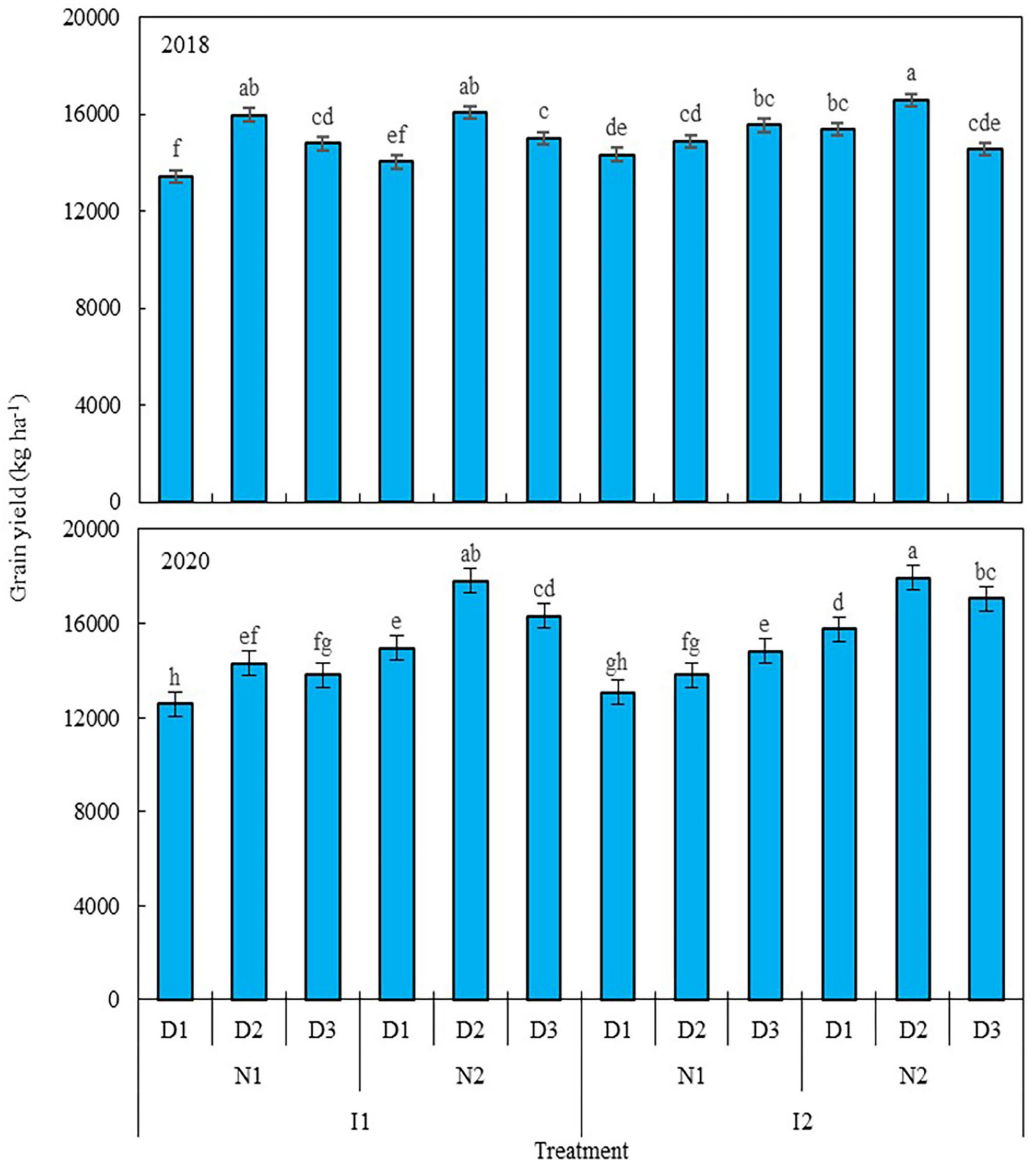
Grain yield of maize as affected by the levels of water, nitrogen, and density in 2018 and 2020. Error bars are standard errors of the means. Different lowercase letters above bars indicate significant differences at the *P* < 0.05 probability level. Descriptions of the irrigation level, nitrogen level, and planting density abbreviations are shown in Table 1.

Nitrogen level and planting density each had a significant effect on the grain yield of maize in 2018 and 2020, as did irrigation × nitrogen level, irrigation × planting density, nitrogen × planting density, and irrigation × nitrogen × planting density interaction terms, but irrigation level did not significantly affect the grain yield of maize. A 25% reduction in nitrogen (N1, 270 kg ha^−1^) decreased the grain yield of maize by 3.1% in 2018 and 21.2% in 2020 (Figure 2), when compared with the traditional nitrogen application level (N2, 360 kg ha^−1^). Compared with low planting density (D1, 7.5 plants m^−2^), using a medium (D2, 9.75 plants m^−2^) and high planting densities (D3, 12 plants m^−2^), increased maize grain yield by 9.5 and 5.7% in 2018, and 9.9 and 8% in 2020, but yields were similar between D2 and D3. At the N1 level, relative to D1, grain yield was increased by 11.1 and 9.2% in 2018 when D2 and D3 were used, and likewise by 9.7 and 11.7% in 2020; for the N2 level, only D2 produced a greater grain yield, increasing by 16.4% in 2018 and 8.7% in 2020, but no significant differences were found between D2 and D3 in either year. Integrating the three factors (irrigation level, nitrogen level, and planting density), using the traditional nitrogen application and medium planting density coupled with a 20% reduction in irrigation or traditional irrigation (I1N2D2 and I2N2D2) resulted in the highest grain yield of maize in this study across the two studied years: it increased by 10.1 and 10.3% in 2018, and by 4.5 and 13% in 2020, in comparison with local traditional irrigation and nitrogen application levels used with the low planting density (I2N2D1). However, no significant difference was found between I1N2D2 and I1N2D3, with the latter entailing a 20% reduction in irrigation coupled with traditional nitrogen application and high planting density. Taken together, these results showed that maize grain yield can be improved by reducing irrigation input and increasing planting density, with the basis of the traditional water and nitrogen input.

### Effects of Different Treatments on Chlorophyll Relative Content

Nitrogen level and planting density each had a significant effect on the chlorophyll relative content (SPAD value) of green leaves for maize in both 2018 and 2020. Furthermore, the value of SPAD was significantly influenced by irrigation × nitrogen level, irrigation × planting density, nitrogen × planting density, and irrigation × nitrogen × planting density interaction terms, but not significantly affected by irrigation level (Table 2). When compared with traditional nitrogen level (N2), in 2018 and 2020, the SPAD value decreased by 18.1 and 17.9%, respectively, with a 25% reduction in nitrogen (N1), and, to a lesser extent, by 7.1 and 4.7%, in the high (D3) density when compared with the low (D1) and medium (D2) treatments in 2018, with no significant difference between D1 and D2 in either year. For the irrigation and nitrogen coupling effect on SPAD value, I1N1 decreased the value of SPAD by 16.9 and 17.9% compared with I1N2, while I2N1 decreased it by 19.3 and 18% compared with I2N2, in 2018 and 2020, respectively. Overall, the I1N2D2 increased the value of SPAD by 7.1% over that in I2N2D1 in 2020, but this significant difference was not found in 2018. Collectively, these results showed that applying a traditional nitrogen fertilizer can induce greater chlorophyll relative content in maize leaves, whose use with rational planting density combined with 20%-reduced irrigation can boost the SPAD value of maize crops in arid irrigation regions.

**Table 2:** SPAD, net photosynthetic rate (Pn), transpiration rate (Tr), and stomatal conductance (Gs) of maize at silking stage as affected by irrigation and nitrogen levels and planting density in 2018 and 2020 at arid irrigated regions.

**Year**	**Irrigation level^a^**	**Nitrogen level**	**Planting density**	**Photosynthetic physiological parameter**			
				**SPAD**	**Pn** **(μmol m^−2^ s^−1^)**	**Tr** **(mmol m^−2^ s^−1^)**	**Gs** **(mol m^−2^ s^−1^)**
2018	I1	N1	D1	54.37^c^^d(b)^	37.98^c^^d^	6.29^c^^d^	0.380^b^^c^
			D2	50.57^ef^	37.49^c^^d^	5.39^f^	0.375^c^
			D3	50.50^ef^	38.07^c^^d^	5.74^e^	0.381^b^^c^
		N2	D1	64.43^a^	42.02^a^	5.84^de^	0.420^a^
			D2	63.80^a^	39.21^b^^c^	6.67^c^	0.392^a^^b^^c^
			D3	58.70^b^	39.65^b^^c^	6.16^d^	0.397^a^^b^^c^
	I2	N1	D1	54.97^c^	40.92^a^^b^	7.96^a^	0.407^a^^b^
			D2	51.53^de^	37.15^d^	8.27^a^	0.371^c^
			D3	48.40^f^	39.20^b^^c^	7.26^b^	0.392^a^^b^^c^
		N2	D1	63.55^a^	39.04^b^^c^	5.36^f^	0.390^a^^b^^c^
			D2	65.55^a^	39.47^b^^c^	5.70^ef^	0.395^a^^b^^c^
			D3	62.93^a^	39.45^b^^c^	6.29^c^^d^	0.394^a^^b^^c^
2020	I1	N1	D1	54.10^f^	38.69^c^^de^	5.43^b^^c^	0.387^b^^c^^d^
			D2	51.43^g^	34.33^f^	5.52^b^^c^	0.343^de^
			D3	51.23^g^	38.77^c^^de^	5.43^b^^c^	0.388^b^^c^^d^
		N2	D1	59.37^e^	43.95^a^	6.53^a^	0.440^a^
			D2	66.57^a^	42.01^a^^b^	6.69^a^	0.420^a^^b^
			D3	64.90^a^^b^	39.29^c^^d^	4.83^c^	0.393^a^^b^^c^
	I2	N1	D1	53.77^f^	41.46^a^^b^	4.99^c^	0.413^a^^b^
			D2	51.80^fg^	37.88^e^	5.87^b^	0.379^b^^c^^d^
			D3	47.80^h^	31.66^g^	5.50^b^^c^	0.317^e^
		N2	D1	62.14^c^^d^	34.61^f^	5.49^b^^c^	0.346^de^
			D2	63.99^b^^c^	40.21^b^^c^	4.08^e^	0.403^a^^b^^c^
			D3	60.87^de^	35.80^f^	4.32^d^	0.358^c^^de^
P-value							
Irrigation level (I)				NS^c^	*	**	**
Nitrogen level (N)				**	*	*	**
Planting density (D)				**	**	*	*
I × N				*	*	**	**
I × D				*	*	*	*
N × D				**	*	*	*
I × N × D				*	*	*	**

a *Treatment abbreviations are described in Table 1*.

b *Within a column for a given year, means followed by different lowercase letters are significantly different at P < 0.05*.

c *NS, no significant difference at the 0.05 probability level*;

** *, significant difference at the 0.05 probability level*;

* *, significant difference at the 0.01 probability level*.

### Effects of Different Treatments on Gas Exchange

#### Net Photosynthetic Rate

Irrigation level, nitrogen level, planting density, and their interactions significantly affected the net photosynthetic rate (Pn) of maize (Table 2). The 20% reduction in irrigation (I1) significantly increased the Pn value by 7% compared with the traditional irrigation level (I2) in 2020, but no significant difference was found in 2018. The Pn of maize in 2018 and 2020, respectively, was 3.4 and 5.5% lower when grown with 25% reduction in nitrogen (N1) level than local traditional nitrogen application level (N2). The high (D3) and medium (D2) planting densities did not differ significantly from the low (D1) planting density in 2018, but in 2020 the D3 decreased Pn by 5.8 and 8.3% compared with D1 and D2, respectively. When cultivated with a 20% reduction in irrigation and medium planting density (I1D2), the Pn value was increased by 4.6 and 22.4% when N2 was used compared with N1. Using the traditional nitrogen application and medium planting density with 20% reduction in irrigation (I1N2D2) increased Pn by 21.4% over I2N2D1 (local traditional irrigation and nitrogen application levels with low planting density) in 2020, but the I1N2D2 and I2N2D1 treatments had similar Pn values in 2018. These results showed that the 20% reduction in irrigation coupled with traditional nitrogen and a moderate density was capable of increasing Pn, and that the photosynthetic capacity of maize was enhanced more by I1N2D2 than the traditional treatment of I2N2D1.

#### Transpiration Rate

Irrigation level, nitrogen level, and planting density had significant effects on the transpiration rate (Tr) of maize leaves, along with their interaction terms (Table 2). The I1 level significantly decreased Tr by 11.6% when compared with I2 in 2018, but significantly increased it by 13.9% in 2020. The N1 level increased Tr by 13.5% relative to the N2 level in 2018, but no significant difference was detected in 2020. Using the D3 decreased Tr by 9.4 and 10.5% when respectively compared with D1 and D2 in 2020, but Tr was not significantly affected by planting density in 2018. At the N2 level, compared with I2, using the I1 treatment decreased Tr by 7.7 and 30% in 2018 and 2020, respectively. The N1 treatment decreased Tr by 6.7 and 9.3% compared with N2 at the I1 level, but the N1 increased Tr by 35.4 and 17.8% relative to N2 when an I2 irrigation level was used to grow maize. Across the irrigation and nitrogen application levels, D2 and D3 had no significant effect on Tr, but the I1N2D2 treatment increased the Tr value by 24.5 and 22% compared with I2N2D1.

#### Stomatal Conductance

The stomatal conductance of the maize leaves was significantly affected by irrigation level, nitrogen level, planting density, and the interactions among the three factors (Table 2). The stomatal conductance (Gs) was increased by 7% in I1 over I2 in 2020, but no significant difference was found in 2018. Compared with N2, the N1 treatment reduced the Gs of maize by 3.4 and 5.5%. Under D3, Gs was reduced by 5.8 and 8.3% vis-à-vis D1 and D2 in 2020, respectively, but it was not significantly affected by planting density in 2018. At the N2 level, the I1 treatment decreased Gs by 13.2% compared with I2 in 2020, but no significant difference was found in 2018. It was found that the I1N2D2 treatment increased Gs by 21.4 and 6.9% over the I2N2D1 and I1N2D3 treatments in 2020, but no significant differences were found in 2018. These results showed that the 20%-reduced irrigation and traditional nitrogen application combined with a rational planting density could provide a promising cropping system to increase the Gs of maize leaves.

### Effects of Different Treatments on Chlorophyll *a* Fluorescence

#### Maximal Photochemical Efficiency of PSII (Fv/Fm) and Actual Photochemical Efficiency of PSII in Light Y(II)

In this study, irrigation level, nitrogen level, and planting density had no significant impact individually on the maximal photochemical efficiency of PSII (Fv/Fm) in 2018 and 2020 (Table 3). This implied that no photo-degradation occurred for the Fv/Fm value in maize ear leaves of plants grown under different irrigation and nitrogen level conditions, and at various densities.

**Table 3:** Maximal photochemical efficiency of PSII (Fv/Fm), actual photochemical efficiency of PSII (Y(II)), dissipation of excess energy (NPQ), quantum yield of regulated non-photochemical energy loss (Y(NPQ)), and quantum yield of non-regulated non-photochemical energy loss (Y(NO)) of maize at silking stage as affected by irrigation and nitrogen levels and planting density in 2018 and 2020 at arid irrigated regions.

**Year**	**Irrigation level^a^**	**Nitrogen level**	**Planting density**	**Chlorophyll a fluorescence parameter**				
				**Fv/Fm**	**Y(II)** **(μmol m^−2^ s^−1^)**	**NPQ** **(mmol m^−2^ s^−1^)**	**Y(NPQ)** **(mol m^−2^ s^−1^)**	**Y(NO)**
2018	I1	N1	D1	0.776^a^^(b)^	0.372^a^^b^	1.119^c^^d^	0.332^c^^d^	0.297^d^
			D2	0.772^a^	0.319^de^	1.124^c^^d^	0.360^a^^b^	0.321^c^
			D3	0.790^a^	0.305^e^	0.932^ef^	0.335^c^^d^	0.360^a^
		N2	D1	0.760^a^	0.366^a^^b^	1.281^a^^b^	0.356^a^^b^	0.278^f^
			D2	0.772^a^	0.389^a^	1.326^a^	0.348^b^^c^	0.263^g^
			D3	0.757^a^	0.370^a^^b^	0.873^f^	0.293^e^	0.336^b^
	I2	N1	D1	0.755^a^	0.379^a^	1.190^b^^c^	0.338^c^^d^	0.284^ef^
			D2	0.763^a^	0.384^a^	1.122^c^^d^	0.326^d^	0.290^de^
			D3	0.776^a^	0.339^c^^d^	0.937^ef^	0.320^de^	0.341^b^
		N2	D1	0.780^a^	0.352^b^^c^	1.352^a^	0.372^a^	0.276^f^
			D2	0.777^a^	0.373^a^^b^	1.282^a^^b^	0.352^b^^c^	0.275^f^
			D3	0.777^a^	0.333^c^^d^	0.993^de^	0.332^c^^d^	0.335^b^
2020	I1	N1	D1	0.794^a^	0.326^e^	1.308^c^	0.382^a^	0.292^d^
			D2	0.792^a^	0.327^e^	1.042^e^	0.343^c^^d^	0.329^a^
			D3	0.794^a^	0.346^d^	1.139^d^	0.348^b^^c^^d^	0.306^c^
		N2	D1	0.765^a^	0.381^b^	1.434^a^^b^	0.364^a^^b^^c^	0.254^g^
			D2	0.780^c^	0.370^b^^c^	1.476^a^	0.376^a^	0.255^g^
			D3	0.788^a^	0.408^a^	1.048^e^	0.303^fg^	0.289^de^
	I2	N1	D1	0.772^a^	0.412^a^	0.954^f^	0.287^g^	0.301^c^^d^
			D2	0.794^a^	0.356^c^^d^	0.966^f^	0.316^ef^	0.327^a^^b^
			D3	0.778^a^	0.374^b^^c^	0.954^f^	0.306^fg^	0.320^a^^b^
		N2	D1	0.787^a^	0.358^c^^d^	1.346^b^^c^	0.368^a^^b^	0.274^f^
			D2	0.765^a^	0.405^a^	1.127^d^	0.315^ef^	0.280^ef^
			D3	0.785^a^	0.348^d^	1.062^de^	0.335^de^	0.317^b^
P-value								
Irrigation level (I)				NS^(c)^	NS	**	**	*
Nitrogen level (N)				NS	**	*	**	**
Planting density (D)				NS	*	**	**	*
I × N				NS	*	*	**	**
I × D				NS	*	*	**	NS
N × D				NS	**	**	**	*
I × N × D				NS	*	**	**	*

a *Treatment abbreviations are described in Table 1*.

b *Within a column for a given year, means followed by different lowercase letters are significantly different at P < 0.05*.

c *NS, no significant difference at the 0.05 probability level*;

** *, significant difference at the 0.05 probability level*;

* *, significant difference at the 0.01 probability level*.

The actual photochemical efficiency of PSII in light [Y(II)] was significantly influenced by nitrogen level, planting density, and the interactions between them, as well as those among irrigation level, nitrogen level, and planting density, but not by irrigation level alone (Table 3). A 25% reduction in nitrogen (N1) significantly decreased Y(II) by 4 and 5.6% compared with the traditional nitrogen application level (N2) in 2018 and 2020, respectively. The maize leaves had higher Y(II) at medium (D2) and high (D3) planting densities than that that at low (D1) density, with D2 notably better in this respect. These results showed that maize grown at the D2 density exhibited a more favorable degree of photo-adaptation. Regarding the effects of irrigation and nitrogen coupling, the value of Y(II) value was 6.3 and 4.4% greater when using a 20% reduction in the irrigation amount (I1) than the traditional irrigation treatment (I2), under the N2 application level, in 2018 and 2020, respectively. Traditional nitrogen application and a medium planting density with a 20% reduction in irrigation (I1N2D2) increased the value of Y(II) by 10.3 and 3.4% compared with traditional irrigation and nitrogen application levels with low planting density (I2N2D1) in 2018 and 2020, respectively. Altogether, these results showed that planting at a rational density combined with 20%-reduced irrigation and traditional nitrogen use can strengthen the photochemical efficiency of the PSII of maize in light in arid irrigation regions.

#### Dissipation of Excess Energy

The dissipation of excess energy (NPQ) of maize leaves was significantly influenced by irrigation level, nitrogen level, planting density, and interactions among the three factors (Table 3). When compared with I2, the I1 treatment increased the NPQ by 16.2% in 2020, but no significant difference was observed in 2018. Conversely, compared with N2, the N1 treatment significantly decreased the NPQ by 9.6 and 15.1%, respectively, in 2018 and 2020. Similarly, The NPQ of maize leaves was 30 and 9.7% higher at D2 than D3, yet similar between D1 and D2, thus indicating that maize grown at medium planting density incurred greater dissipation of excess energy. At the N2 application level, the amount of irrigation amount had no significant effect on the NPQ of the maize leaves. In both years, I1N2D2 significantly increased the NPQ by 9.6% relative to the I2N2D1 treatment in 2020, but no significant difference was found in 2018. These results showed that water and nitrogen management practices can promote the remaining energy dissipation of photosystem II in densely planted maize crops.

#### Quantum Yield of Regulated Non-photochemical Energy Loss

Irrigation level, nitrogen level, planting density, and the interactions among the three factors had significant effects on the quantum yield of regulated non-photochemical energy loss [Y(NPQ)] (Table 3). In 2020, the Y(NPQ) of maize under I1 was 9.8% more than that under I2, and it was 4% higher with N1 than N2, but no significant differences were observed in 2018. With respect to planting density effects, D2 increased the value of Y(NPQ) by 8.3 and 4.5% over D3, in 2018 and 2020, respectively, but it was similar between the D1 and D2 densities. At the I1 irrigation level, the Y(NPQ) value was not significantly different between the N1 and N2 treatments. Among all the treatments, compared with I1N2D3, using I1N2D2 increased the value of Y(NPQ) by 18.8 and 24% in 2018 and 2020, respectively, and increased it by 6.4% over the I2N2D1 treatment in 2018, with no significant difference observed in 2020. These results indicated that using low irrigation and traditional N application integrated with a medium planting density can maintain high Y(NPQ); hence, the I1N2D2 treatment is able to enhance the photo-protection of PSII of the leaves of maize cultivated under arid conditions.

#### Quantum Yield of Non-regulated Non-photochemical Energy Loss

The non-regulated non-photochemical energy loss [Y(NO)] of the maize leaves was significantly influenced by irrigation level, nitrogen level, planting density, and the interactions among the three factors (Table 3). In 2020, Y(NO) was decreased by 5.2% in I1 compared with I2, but no significant difference was found in 2018. Compared with N2, in 2018 and 2020, N1 significantly reduced Y(NO) by 7.4 and 12.4%, respectively. Furthermore, under the D3 density, Y(NO) was 18.9 and 12.5% greater than under D1, and likewise 15.9 and 6.2% greater under D2, in 2018 and 2020, respectively. Conversely, the I1 treatment reduced Y(NO) by 8.4% compared with I2 at the N2 application level in 2020, yet no significant difference was found in 2018. The integrated treatment consisting of I1N2D2 significantly decreased Y(NO) by 4.6 and 6.9% compared with I2N2D1, and likewise by 21.8 and 11.9% compared with I1N2D3. These results showed that when compared with the traditional cropping system (I2N2D1), using the 20% reduction in irrigation combined with traditional nitrogen use and the medium level of planting density (I1N2D2) can reduce photo-damage to PSII in leaves of maize cultivated under arid conditions.

### Principal Component Analysis of Grain Yield, Chlorophyll Contents, Gas Exchange and Chlorophyll Fluorescence Parameters

**Figure 3:**
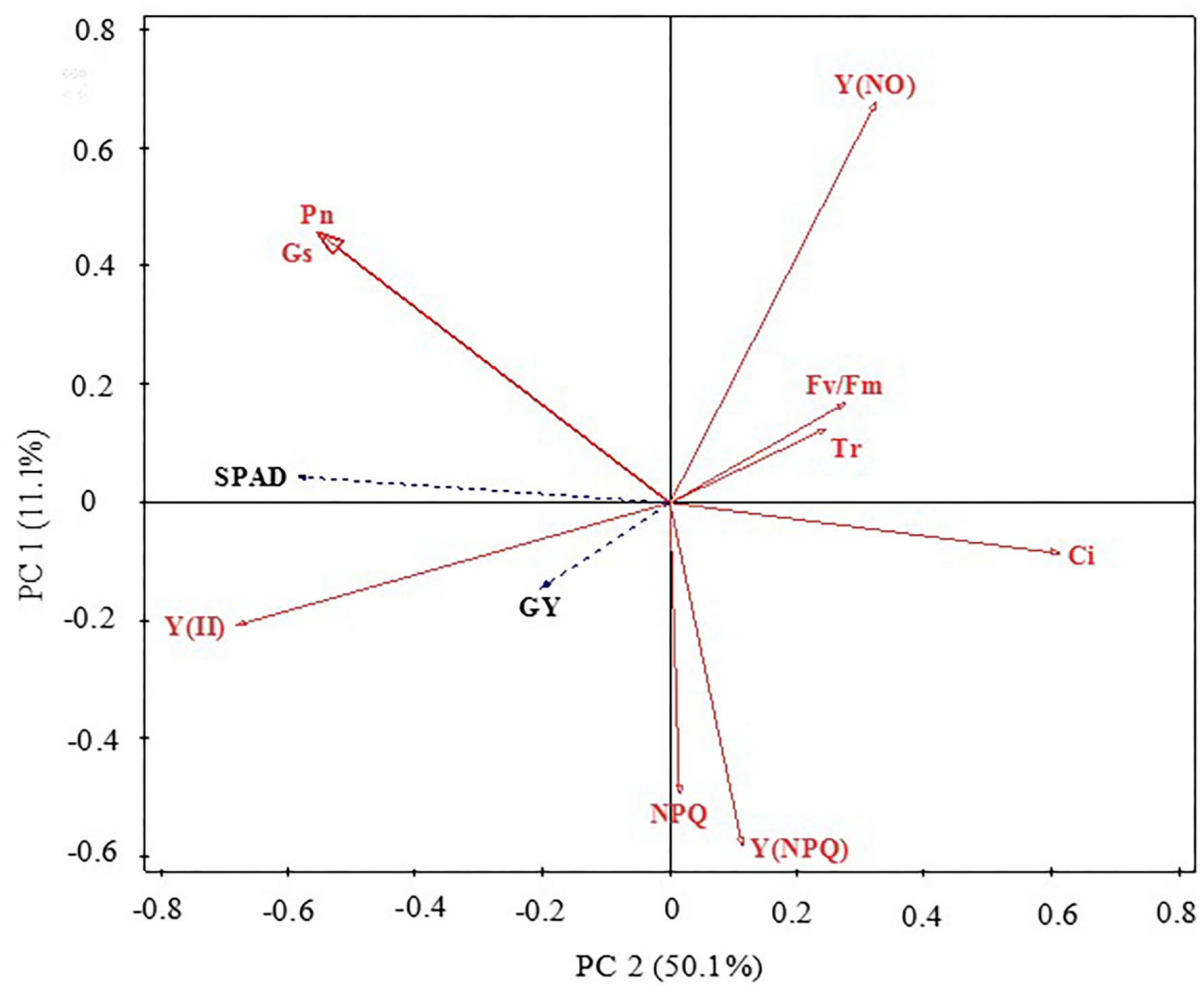
Principal component analysis of maize grain yield, leaf chlorophyll contents (SPAD), gas exchange, and chlorophyll fluorescence parameters. GY, grain yield; SPAD, relative content of chlorophyll; Pn, photosynthetic rate; Gs, stomatal conductance; Tr, transpiration rate; Ci, intercellular CO_2_ concentration; Fv/Fm, maximal photochemical efficiency of PSII; Y(II), actual photochemical efficiency of PSII; NPQ, dissipation of excess energy; Y(NPQ), quantum yield of regulated non-photochemical energy loss; Y(NO), quantum yield of non-regulated non-photochemical energy loss.

Redundancy analysis ordination plots were prepared to represent the relationships between the grain yield, chlorophyll content, and gas exchange and chlorophyll *a* fluorescence parameter factors (Figure 3). The results showed that grain yield, SPAD, and Pn were positively correlated with each other. Overall, the chlorophyll *a* fluorescence and gas exchange parameter factors accounted for 61.2% of the variation in grain yield and chlorophyll content. The distribution direction and characteristics of each parameter can indicate the relationship between parameters. SPAD, Gs, and Pn had strong negative correlations with PC1 but had positive correlations with PC2, suggesting that these changes could represent the photosynthetic capacity of maize leaves. Y(NO), Fv/Fm, and Tr displayed strong positive correlations with PC1 and PC2; both Y(NPQ) and NPQ had a strong negative correlation with PC2, as did Y(II) and GY with PC1 and PC2. These findings further highlight the internal relationships among the key leaf gas exchange parameters, chlorophyll *a* fluorescence parameters, and the grain yield of maize.

## Discussion

### Yield Response

Internal genetic and external environmental factors substantially influence crop yield. Both water and nitrogen are important factors that directly affect the growth and development of crops, either as resources or as resource regulators. Aboveground dry matter accumulation is the main way to obtain a high grain yield from crops (Chen et al., 2015). The proportion of photosynthate stored in leaves and stems of crops is relatively small, and most photosynthetic products are concentrated in grains during the grain filling stage (Chen et al., 2015). Dense planting is an effective measure to manipulate the growth environment of a crop and promote its dry matter accumulation by increasing its leaf area index (Yang et al., 2010a). This study demonstrates that reduced irrigation alongside traditional nitrogen fertilizer use with a medium density of planting can increase the grain yield of maize.

Previous research has indicated that a suitable planting density is an effective and promising way to achieve a high grain yield (Hashemi et al., 2005). Meanwhile, the close planting of individuals increases their leaf area index and could improve the photosynthetic rate of the maize canopy layer (Li et al., 2015). However, an excessive planting density can diminish the photosynthetic traits of the green leaves of each plant, thereby affecting overall crop yield (Li et al., 2014). A study has shown that reduced photosynthetic capacity is closely related to root competition for mineral nutrients (Jensen et al., 2011). The proper application of water and nitrogen weakens the competitiveness of crops and is the key to optimizing dry matter accumulation in crops, photosynthetic capacity, and yield formation (Ercoli et al., 2008). Dry soil conditions severely decrease the supply of mobile ions (nitrate) to the root system and hinder the conversion of soil nutrients into forms available to plants, but excessive irrigation conditions severely increase the loss of mobile ions (e.g., nitrate) and hinder dry matter accumulation (Wolfe et al., 1988). Decreased irrigation water did not reduce the grain yield of maize, because it did not result in the drying of soil and reduced the leaching of nitrate below the root zone; this likely ensured a sufficient nitrogen supply, delayed root senescence, and promoted photosynthesis (Hu et al., 2008). In addition, C_4_ crops can adapt to the environment by reducing stomatal conductance, increasing the absorption rate of CO_2_, and increasing the concentration of CO_2_ around ribulose 1,5-bisphosphate (RuBP) and energy dissipation defensiveness after proper amount of irrigation (Xu, 1988; Zhu et al., 2010). The grass-roots leaves of densely planted maize are weakened by light, resulting in a decline in photosynthetic capacity, but densely planted maize is combined with higher nitrogen fertilizers, and the protein content of RuBP and other enzymes remains at a high level (Xu, 1988; Li et al., 2015). However, the reduced amount of nitrogen application cannot support a high density, because it reduces the photosynthetic key enzyme protein, which is not conducive to improving the photosynthetic capacity of the population and reducing the accumulation of dry matter (Xu, 1988; Zhu et al., 2010). This study also showed that using a high plant density reduced the grain yield, primarily because it decreases photosynthesis per plant (Table 2). This decrease in photosynthesis was driven by reduction in light interception intensity caused by the high planting density of maize (Li et al., 2014). The appropriate application of nitrogen can increase the net photosynthetic rate, transpiration rate, and value of SPAD (Li et al., 2015). It can also improve the ability of mesophyll cells to assimilate CO_2_ to fulfill the potential of dry matter production and increase the dry matter accumulation and grain filling rates in the late growth stage while also promoting organic matter accumulation, which is beneficial for increasing the per capita grain weight.

### Potential Effects of Water and Nitrogen Coupling on the Systematic Regulation of Photosynthetic Characteristics of Maize Under High Planting Density

Water stress can disrupt the growing state of plants, which regulates their metabolic and defense systems to adapt to their ecological environment. Research has shown that applying severe water stress to plants can damage the photosynthetic organs in green leaves (Souza et al., 2004). Water deficit can inhibit the photosynthesis of plants mainly because of augmented stomatal resistance under drought stress conditions, which limits the diffusion of CO_2_ from the air into green leaves (Lawlor, 2002; Lavinsky et al., 2015). In the field experiment, however, the results suggested that the reduced 20% irrigation did not harm photosynthesis in the green leaves of maize, likely because the level of irrigation did not reduce the water demand of maize during the water-sensitive period in either year of the study.

Earlier studies have shown that densely planted plants will inevitably shade each other, thereby limiting photosynthesis (Li et al., 2014). This effect reflects a complicated regulatory network, one that includes leaf structure, photosynthetic electron transport, CO_2_ uptake and transport, assimilation power generation, and the expression levels of Calvin cycle-related enzymes (Taylor et al., 2010; Allen et al., 2011). When individual crops are planted too close to each other, the leaves in the lower part of their canopy will be heavily shaded, and the accompanying reduction in light interception will affect their chlorophyll content, net photosynthetic rate, and stomatal conductance (Makoi et al., 2010), and accelerate leaf senescence (Li et al., 2015). Interestingly, the net photosynthetic rate, transpiration rate, and stomatal conductance of ear leaves also decreased with the increase in planting density (Li et al., 2015). As mentioned above, altered light conditions can induce leaf senescence (Brouwer et al., 2012); accordingly, to some extent, the lowered chlorophyll relative content and net photosynthetic rate in maize green leaves under dense-planting conditions could also reflect the senescence of leaves (Li et al., 2015). Other studies found that the weak light environment in the lower part of densely planted crops affected the photosynthetic capacity of newly expanded (young) leaves in the upper canopy part of populations (Jiang et al., 2011). In contrast to a previous study, the decrease in both Pn and Gs for the maize leaves in this study was relatively small as the maize planting density increased in the field. This is because all the leaves except the new ones were placed in a uniformly low-light environment in that study (Jiang et al., 2011), whereas under realistic growing conditions in this experiment the environmental light intensity at each leaf increases gradually from the bottom to top of the plants. This steady upward increment in incident light intensity may have weakened the systemic regulation of photosynthesis in newly developed leaves in the upper canopy of maize. It is known that the light environment impacts chloroplast pigments and Rubisco content *via* the regulation of leaf nitrogen content, which in turn, affects photosynthetic capacity (Frak et al., 2001). In previous studies, applying nitrogen has been able to significantly increase Pn and SPAD under water deficit conditions (Barton and Colmer, 2006; Wang et al., 2012), simultaneously enhancing the enzymatic actively and content of Rubisco and antioxidant enzymes in leaves (Llorens et al., 2003). Nevertheless, water and nitrogen can also interact to alter stomatal activity. The high solute potential of maize plants receiving adequate nitrogen application promoted their maintenance of turgor pressure and stomatal conductance under water stress (Wolfe et al., 1988). A faster leaf net photosynthetic rate may explain why a moderate nitrogen application increases the nitrogen content of green leaves (Ding et al., 2005). Thus, we speculate that reduced irrigation in tandem with a traditional nitrogen application significantly improves the adaptability of maize to denser planting.

### Potential Effects of Water and Nitrogen Systemic Regulation on Chlorophyll *a* Fluorescence of Maize Under High Planting Density

Chlorophyll *a* fluorescence is a reaction for internal characteristics compared with gas exchange factors. It serves as a powerful tool for the sensitive, rapid, and non-destructive measurement of photochemical and non-photochemical processes (Demmig-Adams et al., 2014). Furthermore, the maximal photochemical efficiency of PSII (Fv/Fm) has become a standard indicator for assessing whether and to what extent leaves suffer from photoinhibition that is based on saturation pulse mode (Peng et al., 2017). In this study, the Fv/Fm of maize was not significantly altered under the tested irrigation, nitrogen, and density levels, indicating that photoinhibition was negligible. However, the actual photochemical efficiency of PSII in light [Y(II)] was increased by traditional nitrogen and medium density management practices, which suggests that these measures, in combination, can delay leaf senescence and maintain high rates of photosynthesis.

Nitrogen has been implicated in the promotion of the photosynthetic rate of leaves in many crops (Li et al., 2013). Alternatively, nitrogen application can increase the transpiration rate under close planting conditions, leading to more xylem-mediated movements of nitrate and cytokinin into leaves (Frak et al., 2001). Normally, leaf senescence is accelerated in partially shaded plants in dense stands of crops, thus reducing photosynthesis (Keech et al., 2007). Nitrogen is known to promote photosynthesis by increasing chlorophyll content and enhancing enzyme activity (Li et al., 2015). However, Y(II) is affected not only by the content of chloroplasts in measured leaves but also by the photochemical activity of the photosynthetic apparatus (Silva-Cancino et al., 2012). Dissipation of excess energy (NPQ), the release of surplus energy mainly *via* heat production, is a vital alternate energy dissipation route in plants (Taiz and Zeiger, 2006; Lambrev et al., 2012). A rise in NPQ with more nitrogen application was observed (Table 3). This increased NPQ, on the one hand, is a photo-protective mechanism; on the other hand, it minimizes the incidence of electron flow at PSI toward the Mehler reaction that reduces oxygen (O_2_) into a superoxide anion (${\text{O}}_{2}^{-}$) (Taiz and Zeiger, 2006). Superoxide anions (${\text{O}}_{2}^{-}$) can further be transformed into various ROS, such as hydroxyl radical (–OH) and hydrogen peroxide (H_2_O_2_), which when overproduced, can cause oxidative damage to cell components (Demidchik, 2015). Finally, leaf Y(NPQ), an indicator of photosynthetic system protection, was increased significantly, whereas Y(NO), an indicator of photosynthetic system damage), was significantly reduced with traditional nitrogen application in maize.

The decline in Y(NO) in traditional nitrogen treatment indicates that the dissipation of maize surplus energy *via* NPQ was adequate; thereby, the damage to photosynthetic system was reduced (Hu et al., 2016; Wu et al., 2019), which is one of the key reasons for the increase of Pn in leaves. In summary, the combination of reduced irrigation water and traditional nitrogen application can properly regulate crop growth and development in the field for adapting to the competition among roots at a suitable density. In this way, the photosynthetic rate, dissipation of excess energy, and regulated non-photochemical energy loss of leaves can be improved, and non-regulated non-photochemical energy loss can be reduced, which is also favorable for the growth and photosynthetic abilities of leaves and contributes to high yields.

The differences in the effects of water and nitrogen interactions on maize grain yield for close-planting can be explained by variations in the gas exchange and chlorophyll *a* fluorescence factors (Figure 3). In addition, SPAD was another major factor that affected the grain yield of maize, although we found that the maize yield was boosted as SPAD increased in arid regions. It is possible that enough chlorophyll relative content during the growth period will increase Pn, which is conducive to dry matter accumulation and grain yield increase for maize.

## Conclusions

Irrigation had no significant influence on the grain yield, gas exchange, and chlorophyll *a* fluorescence of maize leaves. Nitrogen fertilizer application and planting density had a significant influence on the grain yield, gas exchange, and chlorophyll *a* fluorescence of maize leaves. Integrated irrigation, nitrogen, and planting density factors, maize plants grown at low irrigation, traditional high nitrogen with medium density (I1N2D2) had higher net photosynthetic rate (Pn), stomatal conductance (Gs), and transpiration rate (Tr) than these grown at local traditional high irrigation and nitrogen application levels with low planting density (I2N2D1). Moreover, the increased photosynthesis in maize leaves was due to the decrease in Y(NO) (quantum yield of non-regulated non-photochemical energy loss) and increase in Y(II) (actual photochemical efficiency of PSII) and Y(NPQ) (quantum yield of regulated non-photochemical energy loss). Therefore, the I1N2D2 treatment had a greater grain yield by an average of 7.3% than I2N2D1 in the two study years, and it was attributed to the regulation of the photosynthetic function of ear leaves.

## Data Availability Statement

The original contributions presented in the study are included in the article.

## Author Contributions

QC and YG conceived and designed the experiment. YG, QC, and WY performed the statistical analyses. YG,WY, HF, ZF, FH, AY, and CZ were involved in field data collection. QC and WY critically reviewed the manuscript. All authors contributed to writing the paper and approved the final version of the manuscript.

## Funding

This field experiment was supported by the National Natural Science Foundation of China (Nos. 32101857 and 31771738), the Science and Technology Project of Gansu Province (Nos. 20JR5RA025 and 20JR5RA008), the Fuxi Young Talents Fund of Gansu Agricultural University (Gaufx-03Y10), and the Central Government Will Guide Local Science and Technology Development Projects (ZCYD-2020-1-4).

## Conflict of Interest

The authors declare that the research was conducted in the absence of any commercial or financial relationships that could be construed as a potential conflict of interest.

## Publisher's Note

All claims expressed in this article are solely those of the authors and do not necessarily represent those of their affiliated organizations, or those of the publisher, the editors and the reviewers. Any product that may be evaluated in this article, or claim that may be made by its manufacturer, is not guaranteed or endorsed by the publisher.
